# Dermoscopy, reflectance confocal microscopy, and fluorescence staining for the noninvasive diagnosis of crusted scabies

**DOI:** 10.1111/srt.13132

**Published:** 2022-01-16

**Authors:** Li‐wen Zhang, Cong‐hui Li, Xue Shen, Li‐xin Fu, Tao Chen

**Affiliations:** ^1^ Department of Dermatovenereology Chengdu Second People's Hospital Chengdu Sichuan China

**Keywords:** dermoscopy, fluorescence staining, noninvasive diagnosis, reflectance confocal microscopy, scabies

## Abstract

A 91‐year‐old woman presented with a 3‐month history of [extensive](javascript:;) cutaneous lesions with intense pruritus. She lived in a nursing home for a long time. Physical examination revealed a generalized erythematous and scaly rash with intense hyperkeratotic lesions on the neck, trunk, and limbs. Dermoscopy showed a sinuous burrow filled with white dot eggs and feces on the hand with a mite at the end of the burrow. Reflectance confocal microscopy (RCM) manifested a sinuous burrow and a mite. The presence of mites was confirmed with fluorescence staining. The patient was diagnosed with crusted scabies and started treatment with 10% sulfur ointment. Her lesions and pruritus were resolved after 2 weeks.

To the Editor:

A 91‐year‐old woman presented with a 3‐month history of extensive cutaneous lesions with intense pruritus. She lived in a nursing home for a long time. Physical examination revealed a generalized erythematous and scaly rash with intense hyperkeratotic lesions on the neck, trunk, and limbs (Figure 1A and B). Dermoscopy showed a sinuous burrow filled with white dot eggs and feces on the hand with a mite at the end of the burrow (Figure 1C). Reflectance confocal microscopy (RCM) manifested a sinuous burrow and a mite (Figure 1D). The presence of mites was confirmed with fluorescence staining (Figure 1E). The patient was diagnosed with crusted scabies and started treatment with 10% sulfur ointment. Her lesions and pruritus were resolved after 2 weeks.

**FIGURE 1:**
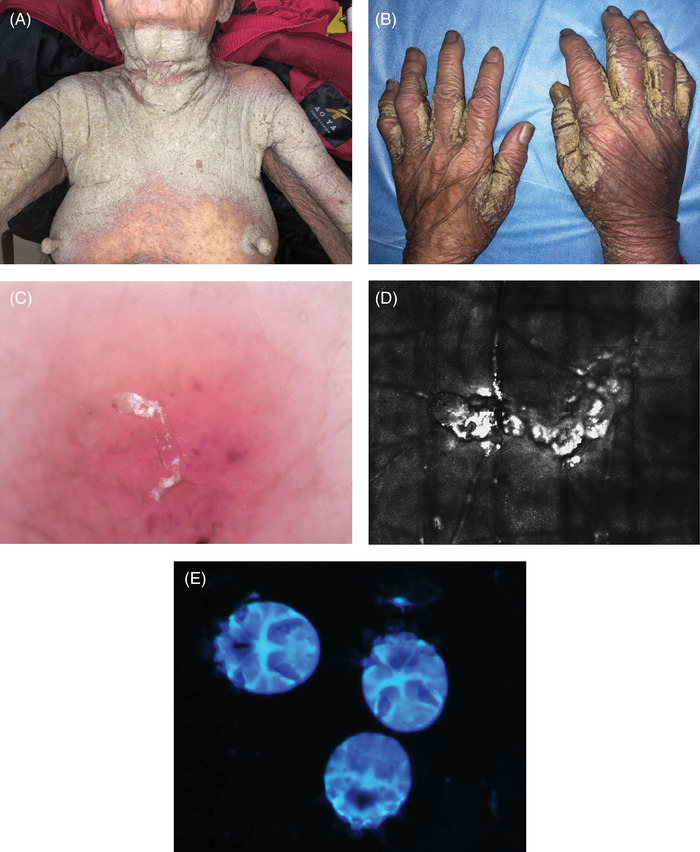
(A) Erythematous plaques with hyperkeratosis on the neck, trunk and arms. (B) The thick scale and deep fissures on the fingers. (C) Dermoscopy showed a sinuous burrow filled with many white dot eggs and feces and an oval translucent mite at the end of the burrow. (D) RCM manifested a sinuous burrow and a mite. (E) Under a fluorescent microscope, many mites with blue‐fluorescence were confirmed

Crusted scabies is a rare and severe form of scabies, characterized by the infestation of up to millions of mites and the development of hyperkeratotic skin crust. Crusted scabies predominantly develop in the patients with immune deficiency, sensory or motor neuropathy, or dementia. Skin lesions commonly present with erythematous plaques that quickly develop tan crusts that predominate in palms, soles, extensor surfaces, and under fingernails. The thick scale and deep fissures on the fingers is considered a distinctive feature.^1^

Under dermoscopy, it is possible to observe a sinuous burrow with a brown jet‐shaped triangular structure that is composed of the pigmented head and anterior legs of mite.^2^ Compared with traditional skin scraping and adhesive tape, dermoscopy was a helpful tool for better diagnosis of scabies and exhibited a significantly higher sensitivity and convenience.^2^

Differential diagnosis for crusted scabies includes other causes of erythroderma such as psoriasis, ichthyosis, atopic dermatitis, cutaneous lymphoma, pityriasis rubra pilaris, and severe seborrheic dermatitis.^3^ The recommended management is a combination of topical and oral agents. Topical scabicide therapy includes 5% permethrin cream, 10% sulfur ointment, and keratolytics. Oral ivermectin should be based on the severity of infection.

The patients in this manuscript have given written informed consent to the publication of their case details.

## FUNDING

This article has no funding source.

## CONFLICT OF INTEREST

The authors have no conflict of interest to declare.
